# Osteoarthritis Was Associated With a Faster Decline in Hippocampal Volumes in Cognitively Normal Older People

**DOI:** 10.3389/fnagi.2020.00190

**Published:** 2020-08-14

**Authors:** Xiang Li, Qiaowen Tong, Jianqing Gao, Cailong Liu, Alzheimer’s Disease Neuroimaging Initiative, Yangbo Liu

**Affiliations:** Alzheimer’s Association; Alzheimer’s Drug Discovery Foundation; Araclon Biotech; BioClinica, Inc.; Biogen; Bristol-Myers Squibb Company; CereSpir, Inc.; Cogstate; Eisai Inc.; Elan Pharmaceuticals, Inc.; Eli Lilly and Company; EuroImmun; F. Hoffmann-La Roche Ltd. and its affiliated company Genentech, Inc.; Fujirebio; GE Healthcare; IXICO Ltd.; Janssen Alzheimer Immunotherapy Research & Development, LLC.; Johnson & Johnson Pharmaceutical Research & Development LLC.; Lumosity; Lundbeck; Merck & Co., Inc.; Meso Scale Diagnostics, LLC.; NeuroRx Research; Neurotrack Technologies; Novartis Pharmaceuticals Corporation; Pfizer Inc.; Piramal Imaging; Servier; Takeda Pharmaceutical Company; and Transition Therapeutics; ^1^Department of Neurology, The First Affiliated Hospital of Wenzhou Medical University, Wenzhou, China; ^2^Department of Neurology, Wenzhou People’s Hospital, Wenzhou, China; ^3^Department of Orthopedics, The First Affiliated Hospital of Wenzhou Medical University, Wenzhou, China

**Keywords:** osteoarthritis, Alzheimer’s disease, hippocampal volumes, longitudinal study, normal cognition

## Abstract

**Objective:**

To examine whether osteoarthritis (OA) is associated with a change in adjusted hippocampal volumes (HpVR: hippocampal/intracranial volume × 10^3^) over time among cognitively normal older people.

**Methods:**

We examined the cross-sectional and longitudinal associations of OA with HpVR among individuals with normal cognition (NC) from the Alzheimer’s Disease Neuroimaging Initiative (ADNI) study. At baseline, a total of 372 individuals with NC were included.

**Results:**

In the cross-sectional analyses of baseline data, we did not find a significant relationship between OA and HpVR among individuals with NC. However, in the longitudinal analyses, OA was significantly associated with change in HpVR over time among individuals with NC. Specifically, compared with individuals without OA, those with OA showed a faster decline in HpVR over time when controlling for other potential confounders, including age, educational attainment, gender, and APOE4 genotype.

**Conclusion:**

OA status was significantly associated with a change in HpVR over time among individuals with NC.

## Introduction

Osteoarthritis (OA) is a common and debilitating disease that influences one or more joints of the body (Hunter and Bierma-Zeinstra, 2019). Emerging evidence demonstrates that OA is also a risk factor for cognitive deficits (Huang et al., 2015; Chen et al., 2018; Weber et al., 2019), as nearly 40% of patients with Alzheimer’s disease (AD) have OA (Wang et al., 2018). Further, a previous preclinical study found that OA can accelerate and exacerbate AD pathologies (Kyrkanides et al., 2011), supporting the notion that OA may have a detrimental impact on AD pathogeneses. However, no previous studies have attempted to examine the association of OA with AD from the pathophysiological perspective in living humans. Additionally, it is not clear whether OA is associated with hippocampal volumes among cognitively normal older people. We hypothesized that OA may be associated with a steeper decline in hippocampal volumes, increasing the risk of AD.

In the present study, we first examined the cross-sectional relationship between OA and baseline hippocampal volumes among cognitively normal older people. Second, we further examined the association of OA with changes in hippocampal volumes over time. The findings may help shed some light on the neuropathological mechanisms by which OA increases the risk of cognitive deficits.

## Materials and Methods

### Alzheimer’s Disease Neuroimaging Initiative Study

Longitudinal data used in the present study were extracted from the Alzheimer’s Disease Neuroimaging Initiative (ADNI) database^1^. The primary aim of ADNI has been to investigate whether demographics, neuropsychological markers, serial MRI, positron emission tomography (PET), and other fluid biomarkers can be integrated to measure the progression of MCI and early AD. The ADNI study was approved by the institutional review board at each ADNI center, and informed written consent was obtained from each participant.

### Participants

**FIGURE 1:**
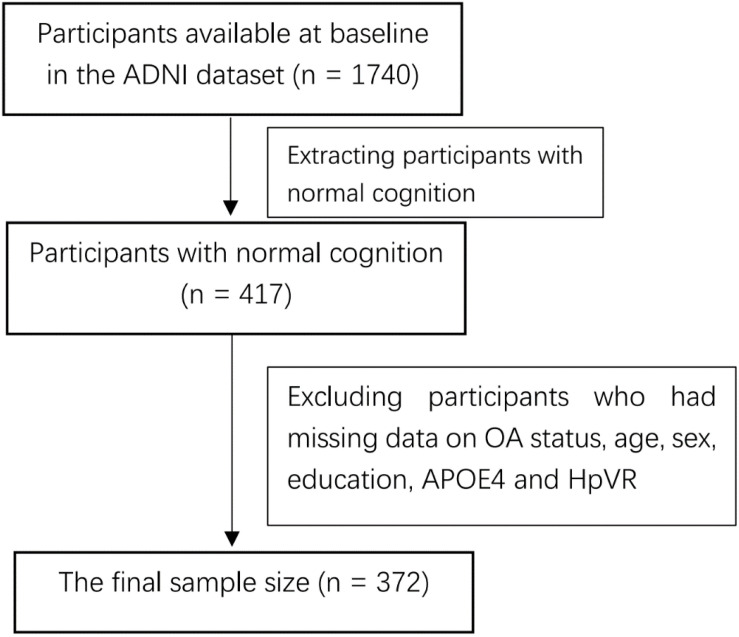
Flowchart of the data selections at baseline.

We included individuals who met the criteria for normal cognition (NC) and had baseline hippocampal volumes data and follow-up measurements of hippocampal volumes. In the present study, there was a total of 372 individuals with NC at baseline. A flowchart of the data selections is presented (Figure 1). Individuals with NC had a clinical dementia rating (CDR) (Morris, 1993) score of 0 and a Mini-Mental State Examination (MMSE) (Folstein et al., 1975) score of 24 or above. Participants with MCI had a CDR score of 0.5, a MMSE score of 24 or higher, objective memory decline as evidenced by the Wechsler Memory Scale Logical Memory II, and an absence of dementia. Patients with AD dementia had an MMSE score of less than 26, a CDR score of less than 0.5, and a diagnosis of probable AD dementia according to the National Institute of Neurological and Communicative Disorders and Stroke and the Alzheimer’s Disease and Related Disorders Association (NINCDS/ADRDA) Criteria. The numbers of individuals present at each follow-up visit are demonstrated in Table 1.

**TABLE 1:** Demographics and clinical variables of the study participants.

**Clinical variables**	**OA− (*n* = 293)**	**OA+ (*n* = 79)**	**Statistic**	***p-*values**
Age, years	74.8 ± 5.36	74.2 ± 6.22	0.75(*t*)	0.56
Female sex, *n* (%)	132 (45.1)	50 (63.3)	8.3 (χ^2^)	0.004
Education, years	16.3 ± 2.8	16.1 ± 2.5	0.62(*t*)	0.54
APOE4 +, *n* (%)	67 (22.9)	30 (38)	7.4 (χ^2^)	0.007
MMSE	29.1 ± 1.1	29 ± 1.2	12176(*w*)	0.45
ADAS-Cog 11	6.08 ± 2.95	5.75 ± 3	0.86(*t*)	0.39
Hippocampal volumes, mm^3^	7350 ± 899	7372 ± 933	−0.19(*t*)	0.85
ICV, cm^3^	1526 ± 158	1488 ± 160	1.9(*t*)	0.06
HpVR	4.85 ± 0.62	4.99 ± 0.66	−1.7(*t*)	0.08
**Follow-up visits, *n* subjects**				
0 year (baseline)	293	79		
1 year	256	74		
2 years	226	62		
3 years	110	22		
4 years	106	18		
5 years	58	8		
6 years	57	13		
7 years	18	3		
8 years	16	1		
9 years	9	0		
10 years	1	0		

*OA: osteoarthritis; MMSE: mini-mental state examination; ADAS-Cog 11: Alzheimer’s disease assessment scale-cognitive 11-item. ICV: intracranial volume; HpVR: hippocampal volume ratio (HpVR, hippocampal/intracranial volume × 10^3^).*

### Hippocampal Volumes

The neuroimaging techniques used by ADNI have been described previously (Jack et al., 2008). Hippocampal volume data were extracted from the ADNI file “ADNIMERGE.csv.” To adjust for gender differences in head size, hippocampal volume ratio (HpVR, formula: hippocampal/intracranial volume × 10^3^) was used as the dependent variable in our models.

### Osteoarthritis Status

The following terms were utilized to screen ADNI subjects’ medical history database: OA and osteoarthritis. We assigned OA status (OA+, OA−) based on participants’ self-reported medical history.

### Statistical Analysis

*T*-test (or Wilcoxon test) and *x*^2^ test were utilized to assess differences in demographics and clinical variables between the two groups (OA+ vs. OA−). To investigate the cross-sectional associations of OA status with MMSE, ADAS-Cog11, and HpVR, *t*-test (ADAS-Cog-11 and HpVT) or Wilcoxon test (MMSE) was performed. To investigate the association of OA status with conversion from NC to MCI or AD dementia, Cox proportional hazard model was performed with adjustments for age and gender. To examine the longitudinal associations of OA status with change in HpVR over time, linear mixed model was fitted for HpVR. This model was adjusted for baseline age, sex, years of education, APOE4 status, and their interactions over time, as well as a random intercept for each participant. The model equation is as following: HpVR-changes ∼ OA × time + age × time + sex × time + education × time + APOE4 × time.

## Results

### Demographical and Clinical Variables

Among individuals with NC, there were 79 participants with OA and 293 participants without OA (Table 1). Participants with OA were more likely to be female (63.3%) compared with those without OA (female 45.1%). In addition, participants with OA were more likely to be APOE4 carriers (38%) compared with those without OA (22.9%). However, there were no significant differences in other variables between the two groups (all *p* > 0.05; Table 1).

### Cross-Sectional Association of OA Status With MMSE, ADAS-Cog11, and HpVR Among Older Individuals With NC

**FIGURE 2:**
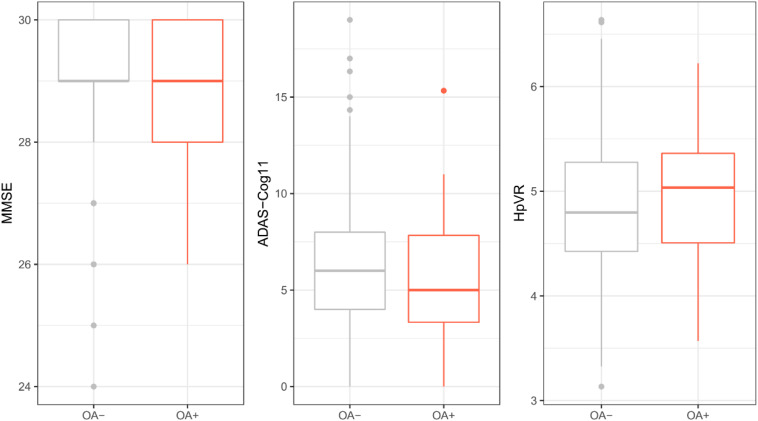
Cross-sectional association of OA with MMSE, ADAS-Cog11, and HpVR among individuals with NC. There was no significant difference in MMSE, ADAS-Cog11, or HpVR between the two groups (all *p* > 0.05). OA: osteoarthritis; MMSE: mini-mental state examination; ADAS-Cog 11: Alzheimer’s disease assessment scale-cognitive 11-item. HpVR: hippocampal volume ratio (HpVR, hippocampal/intracranial volume × 10^3^).

To examine the cross-sectional associations of OA status with MMSE, ADAS-Cog11, and HpVR, *t*-test or Wilcoxon test was conducted. As displayed in Table 1 and Figure 2, among older individuals with NC, there was no significant difference in MMSE, ADAS-Cog11, or HpVR between the two groups (all *p* > 0.05).

### Association of OA Status With Conversion From NC to MCI or AD Dementia

**FIGURE 3:**
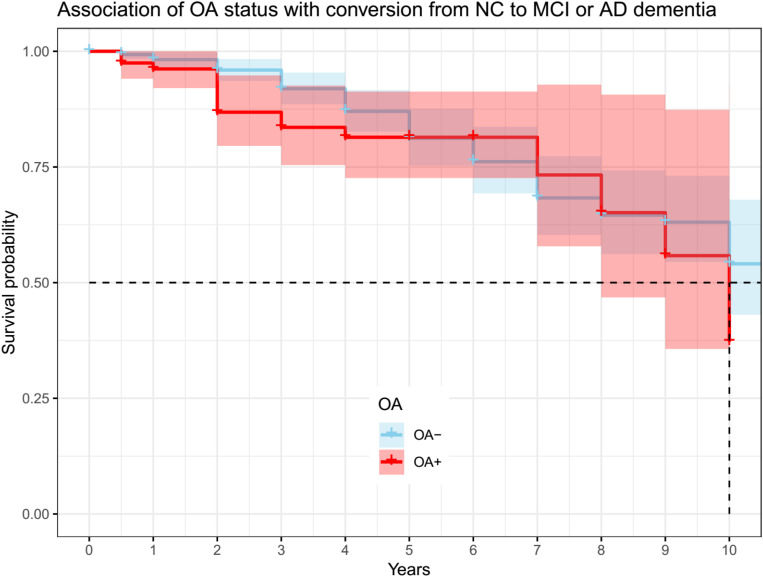
Association of OA status with conversion from NC to MCI or AD dementia. OA status was not associated with conversion from NC to MCI or AD dementia (OA + : HR = 1.6, se = 0.28, *p* = 0.09). OA: osteoarthritis; NC: normal cognition; MCI: mild cognitive impairment; AD: Alzheimer’s disease.

The two groups (OA− vs. OA+) had a similar length of follow-up time (OA− vs. OA + (median (IQR)): 4 (4) vs. 3 (2) years, *p* = 0.07). Cox proportional hazard model was performed to examine the association of OA status with conversion from NC to cognitive impairment (MCI or AD dementia). The model was adjusted for age and gender. However, OA status was not associated with conversion from NC to MCI or AD dementia (OA+: HR = 1.6, SE = 0.28, *p* = 0.09; Figure 3).

### Longitudinal Association of OA Status With Changes in Global Cognition (MMSE and ADAS-Cog 11) Among Older Individuals With NC

Terms reflecting associations with changes in global cognition over time are demonstrated in Table 2. We did not find significant differences in changes in MMSE or ADAS- Cog11 over time between the two groups after adjusting for other potential confounders (Table 2).

**TABLE 2:** Summary of linear mixed models examining the association of OA status with changes in global cognition over time among older individuals with NC.

**Predictors**	**MMSE**			**ADAS-Cog 11**		
	**Estimate**	**SE**	***p-*values**	**Estimate**	**SE**	***p-*values**
Time	0.8185	0.2593	0.0016	0.7718	0.5405	0.1533
OA+	–0.1872	0.1194	0.1168	0.0088	0.3133	0.9776
Age	–0.0158	0.0086	0.0660	0.1045	0.0225	< 0.0001
Female sex	0.2708	0.1009	0.0073	–1.2887	0.2649	< 0.0001
APOE4+	–0.0303	0.1095	0.7819	0.3199	0.2871	0.2652
Education	0.0747	0.0181	< 0.0001	–0.0952	0.0478	0.0464
OA+ × time	0.0009	0.0442	0.9831	–0.0075	0.0921	0.9349
Age × time	–0.0126	0.0031	0.0001	–0.0025	0.0065	0.6968
Female sex × time	0.0223	0.0331	0.5002	0.0006	0.0691	0.9928
APOE4 + × time	–0.0765	0.0367	0.0372	0.2228	0.0766	0.0036
Education × time	0.0028	0.0056	0.6137	–0.0282	0.0117	0.0163

*OA: osteoarthritis; MMSE: mini-mental state examination; ADAS-Cog 11: Alzheimer’s disease assessment scale-cognitive 11-item.*

### Longitudinal Association of OA Status With Hippocampal Atrophy Among Older Individuals With NC

**FIGURE 4:**
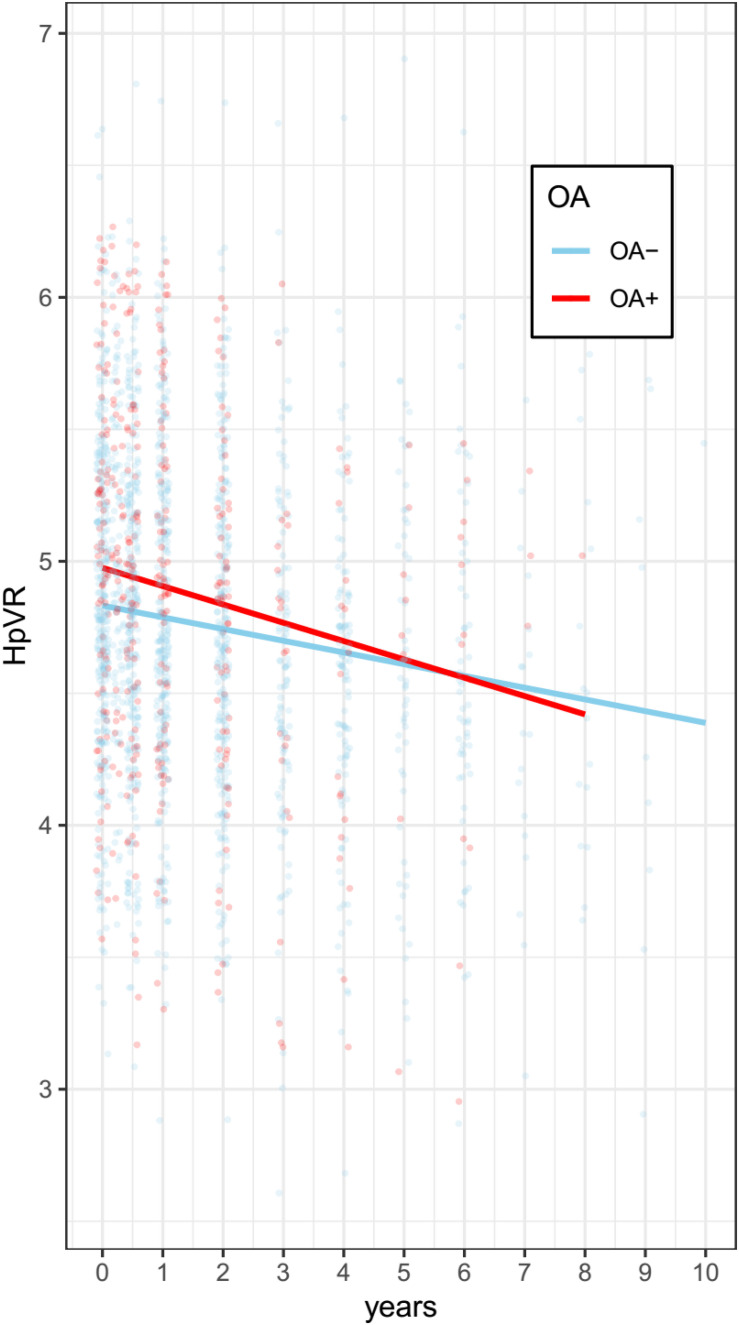
Association of OA with change in HpVR among individuals with NC. Compared with individuals without OA, those with OA showed a significantly steeper decline in HpVR (estimate: –0.0161, *p* = 0.0059) after adjusting for other potential confounders. OA: osteoarthritis; HpVR: hippocampal volume ratio (HpVR, hippocampal/intracranial volume × 10^3^).

Terms reflecting associations with a change in HpVR over time are demonstrated in Table 3. Compared with individuals without OA, those with OA showed a significantly steeper decline in HpVR (estimate: −0.0161, *p* = 0.0059) after adjusting for other potential confounders (Table 3 and Figure 4). Further, we also examined the association of OA status with changes in unadjusted hippocampal volumes (cm^3^) over time among older individuals with NC. We found that OA+ was associated with a faster reduction in unadjusted hippocampal volumes (estimate = −0.0188, SE = 0.0085, *t* value = −2.2031, *p* = 0.0276) after controlling for other covariates.

**TABLE 3:** Summary of linear mixed model examining the association of OA status with changes in HpVR over time among older individuals with NC.

	**HpVR**		
**Predictors**	**Estimate**	**SE**	***p-*values**
Time	0.1336	0.0344	0.0001
OA+	0.0954	0.0673	0.1565
Age	–0.0468	0.0048	< 0.0001
Female sex	0.2226	0.0571	0.0001
APOE4+	–0.0616	0.0615	0.3170
Education	–0.0339	0.0104	0.0011
OA+ × time	–0.0161	0.0059	0.0059
Age × time	–0.0023	0.0004	< 0.0001
Female sex × time	–0.0200	0.0044	< 0.0001
APOE4+ × time	–0.0260	0.0049	< 0.0001
Education × time	0.0004	0.0008	0.5996

*OA: osteoarthritis; HpVR: hippocampal volume ratio (HpVR, hippocampal/intracranial volume × 10^3^). Estimate was unstandardized value, reflecting the magnitude of change in HpVR per year.*

In addition, there were severe drop-offs among individuals after seven years among the two groups. Therefore, the data in the linear mixed model have been limited to seven years for both groups. As shown in Supplementary Table 1 and Supplementary Figure 1, compared with individuals without OA, those with OA showed a significantly steeper decline in HpVR (estimate: −0.015, *p* = 0.0127) after adjusting for other potential confounders.

## Discussion

To the best of our knowledge, this is the first study to examine the cross-sectional and longitudinal associations between OA and adjusted hippocampal volumes (HpVR) among cognitively normal older people. In the cross-sectional analyses, we did not find a significant relationship between OA and HpVR. However, in the longitudinal analyses, there was a significant association of OA with changes in HpVR over time. Specifically, compared with individuals without OA, those with OA showed a steeper decline in HpVR even with adjustment for other covariates.

In the cross-sectional analyses, no significant relationship between OA and baseline HpVR was found among cognitively normal older people. This may be due to the low variability of HpVR in individuals with normal cognition. In the longitudinal analyses, we found that individuals with OA show a steeper decline in HpVR compared with those without OA. This finding was consistent with previous studies, suggesting that OA increases the risk of cognitive impairment (Huang et al., 2015; Chen et al., 2018; Weber et al., 2019). Further, a previous study found that nearly 40% of patients with AD have OA (Wang et al., 2018). In the present study, we found that OA was associated with longitudinal hippocampal atrophy (an important marker of AD) among older individuals with NC, providing critical insight into the neuropathological mechanisms by which OA increases the risk of cognitive deficits.

There are several possible explanations for this finding. One possibility is that both OA and AD involve inflammation. Increasing evidence has suggested that neuroinflammation plays an important role in the pathogenesis of AD (Morales et al., 2014; Heneka et al., 2015; Zhang and Jiang, 2015). The inflammatory response drives microglia to overact and release a variety of inflammatory mediators, such as TNF-α, Il-1, and IL-6, which contribute to cell apoptosis and neuron loss (Block et al., 2007; Querfurth and Laferla, 2010). Similarly, Il-1β and TNF-α are two important proinflammatory cytokines that may be involved in the pathogenesis of OA (Goldring et al., 1996; Westacott and Sharif, 1996; Martel-Pelletier et al., 1999). Additionally, levels of several proinflammatory cytokines in serum were found to be associated with the risk of AD dementia (Tan et al., 2007; Holmes et al., 2009). A previous preclinical study using an AD mouse model found that OA triggers neuroinflammation and subsequently accelerates AD pathology (Kyrkanides et al., 2011). Another possibility is that physical inactivity because of OA may increase the risk of AD and hippocampal atrophy. For instance, a prospective longitudinal study revealed that a higher level of physical activity can reduce the risk of cognitive decline and AD dementia among older individuals without dementia (Buchman et al., 2012). In contrast, in a community-based cohort, higher levels of physical activity were found to be positively related to hippocampal volumes (Tan et al., 2017).

There are several limitations in our study. First, the classification of OA was based on the participants’ self-report, which may yield some misclassifications due to self-report bias. The ADNI study did not apply structured questionnaires for recording participants’ medical history. Therefore, subjects without OA did not have any information about OA. This limitation may also yield some misclassifications due to self-report bias. Second, as shown in Table 1, compared with individuals without OA, those with OA were more likely to carry at least one copy of APOE4 allele, which is also associated with cognitive decline and hippocampal atrophy (Liu et al., 2016). We cannot rule out the possibility that the effect of OA on changes in HpVR may actually be due to the APOE4 genotype, though our model was adjusted for the APOE4 genotype. Third, the sample size of individuals with OA was relatively small. Further studies with larger sample sizes are needed to validate our findings. Fourth, physical inactivity is a potential mechanism by which OA may be associated with cognitive impairment. Further studies should include this variable as a potential covariate in the analysis. Finally, in the cross-sectional analysis, we only observed a marginally significant difference in HpVR between the two groups (*t* = 1.9, *p* = 0.08). However, in the longitudinal analysis, we detected a significant association of baseline OA status with changes in HpVR (estimate = −0.0161, *p* = 0.0059) or unadjusted hippocampal volumes (estimate = −0.0188, *p* = 0.0276) over time. Therefore, our data suggest that OA may be associated with longitudinal hippocampal atrophy.

In conclusion, we found that cognitively normal older individuals with OA show a steeper decline in HpVR compared with those without OA. Our data provide neuropathological evidence regarding the impact of OA that may help explain previously established relationships between OA and cognitive impairment.

## Data Availability Statement

The datasets are publicly available (http://adni.loni.usc.edu).

## Ethics Statement

The studies involving human participants were reviewed and approved by The ADNI study. The patients/participants provided their written informed consent to participate in this study.

## Author Contributions

YL designed and supervised the study. XL, QT, JG, and CL performed the research, analyzed the data, and wrote the manuscript. All authors contributed to the article and approved the submitted version.

## Conflict of Interest

The authors declare that the research was conducted in the absence of any commercial or financial relationships that could be construed as a potential conflict of interest.

## Footnotes

1 http://adni.loni.usc.edu
