# Supplementary material for: Osteoarthritis Was Associated With a Faster Decline in Hippocampal Volumes in Cognitively Normal Older People
Source: Front Aging Neurosci. 2020 Aug 14;12:190. doi: 10.3389/fnagi.2020.00190 (PMC7456859; doi:10.3389/fnagi.2020.00190)
Supplement: Supplementary file 1 [file Image_1.pdf]

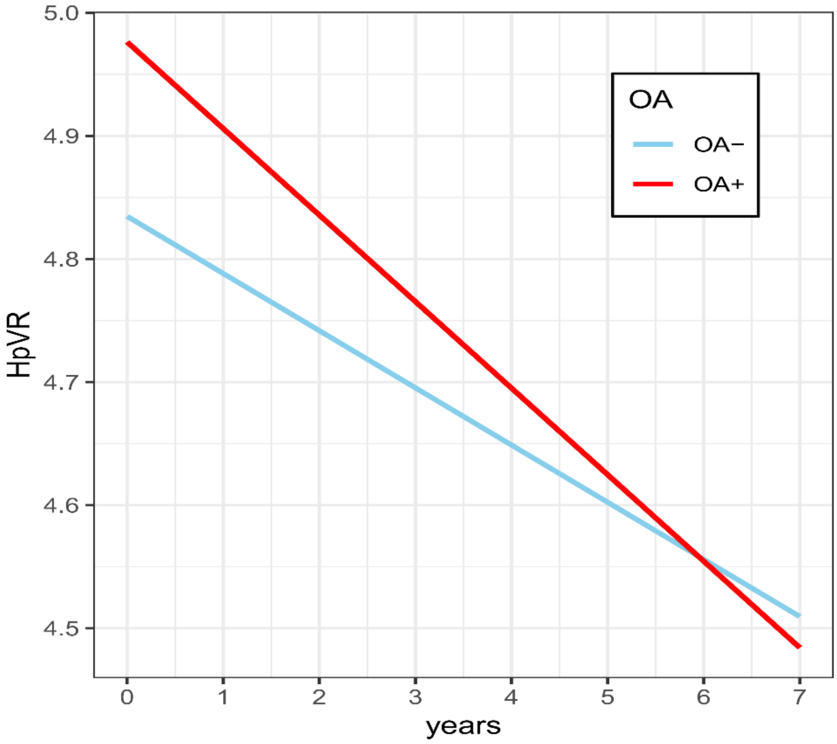

Supplementary Figure 1. Association of OA with change in HpVR among individuals with NC (Limiting the follow-up time to 7 years). Compared with individuals without OA, those with OA showed a significantly steeper decline in HpVR (estimate: -0.015, $p = 0.0127$) after adjusting for other potential confounders. Abbreviations: OA: osteoarthritis; HpVR: hippocampal volume ratio (HpVR, hippocampal/intracranial volume $\times 10^3$).
