# Supplementary material for: Osteoarthritis Was Associated With a Faster Decline in Hippocampal Volumes in Cognitively Normal Older People
Source: Front Aging Neurosci. 2020 Aug 14;12:190. doi: 10.3389/fnagi.2020.00190 (PMC7456859; doi:10.3389/fnagi.2020.00190)
Supplement: Supplementary file 2 [file Table_1.docx]

**Supplementary Table 1. Summary of linear mixed model (limiting the follow-up time to 7 years)**

|  | HpVR | | |
| --- | --- | --- | --- |
| Predictors | Estimate | SE | p values |
| OA+ × time | -0.015 | 0.006 | 0.0127 |
| Age × time | -0.002 | 0.0004 | < 0.001 |
| Female sex × time | -0.025 | 0.005 | < 0.001 |
| APOE4+ × time | -0.02 | 0.005 | < 0.001 |
| Education × time | 0.0007 | 0.0008 | 0.41 |

Abbreviations: OA: osteoarthritis; HpVR: hippocampal volume ratio (HpVR, hippocampal/intracranial volume ×10^3^). Estimate was unstandardized value, reflecting the magnitude of change in HpVR per year.
